# Impact of Ascorbic Acid on the In Vitro Iron Bioavailability of a Casein-Based Iron Fortificant

**DOI:** 10.3390/nu12092776

**Published:** 2020-09-11

**Authors:** Magalie Sabatier, Andreas Rytz, Joeska Husny, Stéphane Dubascoux, Marine Nicolas, Anant Dave, Harjinder Singh, Mary Bodis, Raymond P. Glahn

**Affiliations:** 1 Nestlé Research, Société des Produits Nestlé, 1000 Lausanne, Switzerland; andreas.rytz@rdls.nestle.com (A.R.); stephane.dubascoux@rdls.nestle.com (S.D.); Marine.Nicolas@rdls.nestle.com (M.N.); 2 Nestlé Product and Technological Center, Société des Produits Nestlé, 3510 Konolfingen, Switzerland; Joeska.Husny@rdko.nestle.com; 3 Riddet Institute, Massey University, Private Bag 11222, Palmerston North 4442, New Zealand; a.dave@massey.ac.nz (A.D.); H.Singh@massey.ac.nz (H.S.); 4 United States Department of Agriculture, Agricultural Research Service (USDA/ARS), Robert Holley Center for Agriculture and Health, 538 Tower Road, Ithaca, NY 14853, USA; msb15@cornell.edu (M.B.); rpg3@cornell.edu (R.P.G.)

**Keywords:** iron‒casein complex, ferrous sulfate, micronized ferric pyrophosphate, solubility, dissociation, bioaccessibility, Caco-2 cell culture model

## Abstract

A new iron–casein complex (ICC) has been developed for iron (Fe) fortification of dairy matrices. The objective was to assess the impact of ascorbic acid (AA) on its in vitro bioavailability in comparison with ferrous sulfate (FeSO_4_) and ferric pyrophosphate (FePP). A simulated digestion coupled with the Caco-2 cell culture model was used in parallel with solubility and dissociation tests. Under diluted acidic conditions, the ICC was as soluble as FeSO_4_, but only part of the iron was found to dissociate from the caseins, indicating that the ICC was an iron chelate. The Caco-2 cell results in milk showed that the addition of AA (2:1 molar ratio) enhanced iron uptake from the ICCs and FeSO_4_ to a similar level (*p* = 0.582; *p* = 0.852) and to a significantly higher level than that from FePP (*p* < 0.01). This translated into a relative in vitro bioavailability to FeSO_4_ of 36% for FePP and 114 and 104% for the two ICCs. Similar results were obtained from water. Increasing the AA to iron molar ratio (4:1 molar ratio) had no additional effect on the ICCs and FePP. However, ICC absorption remained similar to that from FeSO_4_ (*p* = 0.666; *p* = 0.113), and was still significantly higher than that from FePP (*p* < 0.003). Therefore, even though iron from ICC does not fully dissociate under gastric digestion, iron uptake suggested that ICCs are absorbed to a similar amount as FeSO_4_ in the presence of AA and thus provide an excellent source of iron.

## 1. Introduction

Iron is an essential micronutrient with well-established contributions to body functions such as the formation of red blood cells and hemoglobin, oxygen transport, cell division, energy metabolism, immunity and cognition [1]. According to the most recent estimates, 25% and 37% of anemia is associated with iron deficiency in preschool children and women of reproductive age, respectively [2]. Food fortification with iron is generally regarded as the most cost-effective and sustainable long-term approach for reducing the risk of iron deficiency [3]. However, adding a bioavailable form of iron in some food products (e.g., with either a high moisture, and/or a low pH, and/or containing fat, polyphenols) in a relevant nutritional quantity remains a technological challenge, as soluble forms of iron such as ferrous sulfate (FeSO_4_) can lead to organoleptic changes. The less soluble forms of iron (e.g., iron pyrophosphate (FePP)) are generally more stable in difficult-to-fortify products but are also less well absorbed. A highly water-soluble iron–casein complex (ICC) with improved organoleptic properties [4,5], i.e., a sodium caseinate‒ferric (Fe^3+^) phosphate complex, has recently been developed. This complex is formed through the interaction of iron with sodium caseinates, in the presence of orthophosphate [6,7]. The reported distribution of added inorganic ferrous or ferric iron in the casein fraction of cow’s milk (i.e., 65–90%; pH 6.5–6.7) suggests that the formation of similar complexes may occur naturally in iron-fortified milk [8].

The absorption of iron from this complex was assessed in young women using stable isotopes and the erythrocyte measurement method [9], and showed no statistical difference to that from FeSO_4_ when added to milk [10]. This clinical trial was performed without the addition of ascorbic acid (AA), which is recognized to be the most effective enhancer of iron absorption and to counteract the effects of most iron absorption inhibitors [3,11]. The most potent inhibitors are phytic acid and polyphenols and, to a lesser extent, calcium and some proteins (e.g., β-lactoglobulin and caseins) [11], both of which are present in milk. Calcium could affect iron absorption by affecting interactions with ligands in the gut lumen and by impairing the uptake of ferrous ion by the divalent metal transporter 1 (DMT1) [12,13]. The first effect would be overcome by AA addition, as shown by studies in milk performed with and without AA [14,15,16]. The second effect would be observed only in single-meal studies [17,18] and would be compensated for over time by physiological adaptation of iron absorption to increased calcium intake, as suggested by reviews [17,19] of long-term studies with dietary [20,21,22,23,24] or supplemental doses of calcium in humans. Approximately 82% of cow’s milk proteins are caseins, which are divided into four subclasses (i.e., α_s1_, α_s2_, β– and κ–). Caseins are known to link strongly to iron through their clusters of phosphoserine residues [25], but these proteins are also reported to bind iron on other amino acid residues with weaker interactions [26]. When linked to phosphoserine clusters, their effect towards iron absorption is reported to depend on the casein type, and on the structure and/or the conformation of fragments (i.e., caseinophosphopeptides) generated during their digestion [27,28]. Only a few studies have reported the effect of caseins from semi-synthetic meals, and their hydrolysis was found to increase iron absorption [29]. To our knowledge, iron absorption from a pure iron‒α-caseinophosphopeptide complex has never been explored. In rats, iron‒β-caseinophosphopeptide complexes exhibited either a similar or a higher iron absorption than from FeSO_4_ and iron gluconate [30,31,32]. In young adult women, the iron absorption from iron‒β-caseinophosphopeptide complexes (1–25) was found to be similar to that from FeSO_4_ when ingested with a glass of milk [33]. In all these studies, the impact of AA on the absorption of iron from iron bound to casein or a fragment (peptide) was not investigated.

The addition of AA to iron-fortified food (e.g., milk, cereals) is recommended by the World Health Organization to optimize iron absorption [3], and the European Food Safety Authority has accepted the functional health claim “ascorbic acid increases iron absorption” [34]. AA can increase the absorption of both ferrous ion (Fe^2+^) and ferric ion (Fe^3+^) [35] in a dose-dependent manner, and its impact would also depend on the solubility of the iron salt used for fortification [35,36,37,38]. Its effect is attributed mainly to its reducing properties [35,37], allowing the iron to stay soluble through a wide range of pH (from 2 to 11) and to be absorbed via the DMT1 in the small intestine [35]. However, AA would facilitate ferric iron absorption by interacting with ferric ion at acid pH [35]. Thus, for AA to exert its effect, iron from the ICC would need to be released from the complex and to remain soluble and exchangeable under gastric conditions in the gut lumen. We hypothesized that AA would increase iron absorption from the ICC, similar to that from inorganic iron salts. To test this hypothesis, in vitro experiments were performed to determine whether iron from the ICC was soluble and whether it dissociated from the caseins under acidic gastric pH; the digestibility of the ICC was also determined. Then, in vitro-simulated digestion coupled with the Caco-2 cell model [39] was used to evaluate whether AA has an impact on the in vitro iron bioavailability of the ICC in comparison with FeSO_4_ and FePP in water and milk. The Caco-2 cell model has the great advantage that it has been validated against human data for the evaluation of the interaction between AA and iron [40]. Based on the data generated, the fate of the ICC during digestion was proposed.

## 2. Materials and Methods

### 2.1. Preparation of the ICC

Two batches of the ICC were produced by the Nestlé Technological Center (Konolfingen, Switzerland) as per the protocol previously described by Mittal et al. [6,7]. The sodium caseinate (Arla Foods Ingredients, Viby J, Denmark) used to produce the complexes contained 36.3% α_s1_-casein, 8.5% α_s2_-casein, 40.7% β-casein and 14.5% κ-casein. Caseinate was dissolved in deionized water and chilled to 4 °C. Ferric and phosphate salts were added at constant pH (pH 7) with vigorous stirring. The final protein solution containing iron was spray dried. The iron contents of the two batches were 2.80 ± 0.07 and 2.91 ± 0.07 mg Fe/100 g powder for batch 1 (ICC1) and batch 2 (ICC2), respectively.

### 2.2. Solubility and Iron Dissociation at Acidic pH

The in vitro solubility of the ICCs was compared with that of FeSO_4_ heptahydrate (20.08% iron, Dr. Paul Lohmann GmbH, Emmerthal, Germany) and a micronized FePP (20.25% iron, Aksell Química, Indaiatuba, Brazil) at pH 1.7 using a protocol adapted from Lynch et al. [41] and Henare et al. [10]. Two aliquots of each iron compound, each containing 20 mg of iron, were weighed into 500 mL conical flasks. All salts were diluted in 250 mL of HCl (pH 1.7) in the flasks, at room temperature. The flasks were placed in a shaking water bath at 37 °C and gently agitated at a rate of 2 Hz for 90 min. Aliquots of 1.5 mL from each flask were taken at 5, 15, 30, 60 and 90 min and centrifuged at 14,000× *g* for 5 min (VWR MicroSTAR 17, Fontenay Sous Bois, France). The iron content was determined in the initial samples and in the supernatants to calculate the percentage of iron solubility over time for each compound. This experiment could not determine whether the iron in the ICC was dissociated from the proteins. Thus, the trial was repeated at pH 2 to evaluate the dissociation of iron from the proteins using size-exclusion chromatography columns (PD-10 desalting columns, Sephadex^TM^ G-25M, GE Healthcare, Chalfont St. Giles, UK). FeSO_4_ was used as a control. Acidic solutions of the ICC were prepared with diluted HCl (pH 2) in duplicate, as described above. At 30 min after the beginning of the experiment, the supernatants were collected after centrifugation (i.e., 14,000× *g* for 20 min) to allow the loading of 2.5 mL on the columns. The experiment was performed using the gravity protocol provided by GE Healthcare instructions 52-1308-00 BB. Diluted HCl (pH 2) was used for the two consecutive elution steps. Volumes of 3.5 and 6 mL were collected after the first and second elutions, respectively. The iron contents in the two eluates, corresponding to the protein fraction and the free mineral fraction, respectively, were analyzed. An aliquot (1 mL) from all samples was diluted to 10 mL with HNO_3_ (8%) and analyzed by inductively coupled plasma‒optical emission spectrometry (Agilent™ 5100 model, Agilent, Santa Clara, CA, USA), according to Association of Official Agricultural Chemists (AOAC) official method 2011.14.

### 2.3. Digestibility and Solubility of the ICC under Simulated Gastric Digestion

The digestibility of the two batches of ICC and sodium caseinate (Arla Foods Ingredients, Viby J, Denmark) was determined using a protocol adapted from the INFOGEST’s static in vitro digestion protocol at Massey University, Palmerston North, New Zealand [42]. Aqueous solutions of the ICCs (1%, *w/v*) were mixed with simulated gastric fluid (pH 2) and pre-incubated to 37 °C for 15 min to allow temperature equilibrium. A stock pepsin solution (10 mg/mL, 3500 U/mg) was added to the mixture to achieve a final activity of 2000 U/mL in the digestion mixture and the digestion was started. Samples were taken at various incubation times (0.5, 5, 10, 20 and 60 min) and were immediately diluted in reducing tricine sodium dodecyl sulfate polyacrylamide gel electrophoresis (SDS-PAGE) sample buffer (pH 8.45, 2% *w/v* SDS and 200 mM dithiothreitol) to obtain a final protein concentration of approximately 1 mg/mL. The samples were then analyzed by tricine‒SDS-PAGE as per the methodology described previously [43]. The gels (49.5% T, 3% C) were prepared in house and 10 μL of each sample was loaded onto the gels. They were scanned using a Molecular Imager Gel Doc XR system (Bio-Rad Laboratories, Hercules, CA, USA) and images were analyzed using QuantityOne software (Bio-Rad Laboratories, Hercules, CA, USA). Undigested samples and pepsin solution were used as controls.

In parallel, a similar protocol of simulated digestion (i.e., pH 2 with the addition of pepsin) was applied to the two batches of ICC to measure the solubility of the products of digestion, as reported above. The iron content was determined in the initial samples and in the supernatants after centrifugation (i.e., 14,000× *g* for 10 min) to calculate the percentage of iron solubility over time (i.e., at 10, 30 and 60 min) for each compound. FeSO_4_ was used as a reference.

### 2.4. In Vitro-Simulated Digestion Coupled with the Caco-2 Cell Model

The impact of AA on the in vitro iron bioavailability from the ICCs was evaluated using the established in vitro-simulated digestion coupled with the Caco-2 cell model at USDA/ARS, Cornell University, Ithaca, NY, USA, as described previously [39,40]. In brief, the food matrix underwent a simulated gastric digestion with pepsin at pH = 2, 37 °C for 1 h to mimic the gastric phase of the digestion in individual above 2 years old. This step was followed by a simulated intestinal digestion with pancreatin and bile at pH = 7, 37 °C for 2 h (*n* = 3) to mimic the duodenal phase. This second step took place on a dialysis membrane placed above the Caco-2 cell monolayers. During the digestion process, iron was released from the food matrix. Solubilized iron can diffuse through the membrane and be taken up by the cells. Thus, in response to higher intracellular iron concentrations, Caco-2 cells will form ferritin. The formation of ferritin was quantified as an indicator of iron uptake by the cells. The Caco-2 cells (obtained at passage 21; American Type Culture Collection, Gaithersburg, MD, USA) were seeded in 6-well collagen-coated plates at passage 29–42. Cells were grown for 13 days before each bioassay at 37 °C in an incubator using Dulbecco’s modified Eagle’s medium supplemented with 25 mM HEPES (pH 7.2), 10% (*v/v*) fetal bovine serum and 1% antibiotic/antimycotic solution. The medium was changed every 2 days. Twenty-four hours prior to each bioassay, the culture medium was replaced with iron-free Minimum Essential Medium (MEM [pH 7]; GIBCO) supplemented with 10 mM PIPES (piperazine-N,N’-bis-[2-ethanesulfonic acid]), 1% antibiotic– antimycotic solution, hydrocortisone (4 mg/L), insulin (5 mg/L), selenium (5 μg/L), triiodothyronine (34 μg/L) and epidermal growth factor (20 μg/L). A fresh 1 mL aliquot of MEM (pH 7) covered the cells during each experiment. Eighteen hours after the start of the experiment, the cells were harvested, after rinsing and sonication of the plates for 15 min. Cells were then scraped from the plate surface and transferred into tubes for analysis. Ferritin was measured by enzyme-linked immunosorbent assay. The results were normalized to the total protein content of the Caco-2 cells and were expressed as ng ferritin/mg protein (*n* = 3). The complex was tested in a milk and water matrices, in comparison with FeSO_4_, the reference compound for iron bioavailability, and with FePP, the main salt used for milk fortification. In water, only ICC (2.8%) was evaluated with and without AA at a molar ratio of AA to iron of 2:1, as a control. In milk, the two ICCs were evaluated with and without AA at molar ratios of AA to iron of 2:1 and 4:1. The milk was a full cream powder containing 26% fat and 875 mg calcium/100 g powder. The amount of iron corresponded to 3.3 mg Fe/serving of milk, i.e., 33 g of powder for 250 mL of milk ready to be consumed. An aliquot (2 mL) of each individual digest, corresponding to approximately 26.4 μg of iron, was loaded onto the dialysis membrane placed on the Caco-2 cell monolayers.

### 2.5. Statistics

Descriptive statistics—namely arithmetic mean and standard deviation (SD)—were used to summarize all outcomes. In the case of in vitro iron bioavailability, the raw ferritin data were first adjusted for protein content and then log-transformed because of their log-normal distribution. These transformed data were then analyzed using two-way analysis of variance to test the significance of main effects (iron sources, AA addition) and their interaction. Since both main effects and their interaction appeared to be highly significant (*p* < 0.01), the Tukey‒Kramer method was used for all subsequent pairwise comparisons of interest, with a significance level of α = 5%. With this method, any two conditions can be declared to be significantly different if their mean difference is larger than the honestly significant difference (HSD5%). This analysis was visualized using a bar chart (means) and error bars (mean ± 0.5 × HSD5%), so that two conditions appeared to be significantly different if their error bars did not overlap [44].

## 3. Results

### 3.1. Solubility and Iron Dissociation at Acidic pH

**Figure 1:**
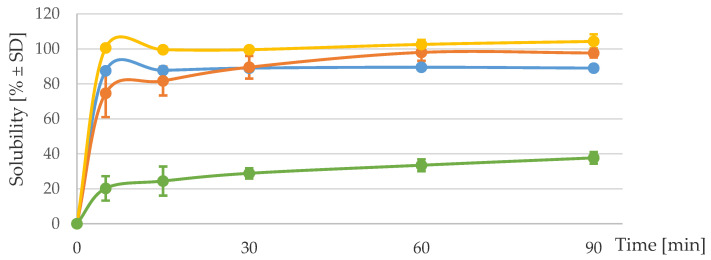
Solubility (% ± Standard deviation (SD)) of iron salts and of iron from the two batches of iron‒casein complex at pH 1.7 over time. The solubilities of ferrous sulfate, micronized ferric pyrophosphate, iron‒casein complex (2.9%), and iron‒casein complex (2.8%) are represented by (-•-), (-•-), (-•-) and (-•-), respectively.

The solubilities of the iron compounds in diluted acid (pH 1.7) are reported in Figure 1. There was a rapid solubilization of iron from the ICCs, i.e., >75 ± 19.3% at 5 min and >89 ± 0.3% at 90 min. The kinetics from the ICCs were similar to that from FeSO_4_. The solubility of FePP remained low over time and reached a maximum solubility of 37.6 ± 4.7% at 90 min.

As caseins are known to be soluble at acidic pH, to evaluate whether the iron compounds dissociated from the proteins under acidic conditions, the soluble fractions of the compounds were eluted through size-exclusion chromatography columns at 30 min after the start of the experiment. As expected, 86.6 ± 2.7% of FeSO_4_, an iron salt that is known to fully dissociate from its counterion under acidic conditions, was retrieved in the second eluate, corresponding to the free mineral fraction. Only 10.7 ± 0.3% of ICC (2.8%) and 20.5 ± 0.3% of ICC (2.9%) were retrieved in this fraction. The percentages of iron appearing in the eluates corresponding to the protein fraction were 77.9 ± 1.9, 55.2 ± 6.7 and 4.7 ± 0.7% for ICC (2.8%), ICC (2.9%) and FeSO_4_, respectively. These data indicate that the majority of the iron from the ICCs remained bound to the protein under acidic conditions. It cannot be excluded that a higher percentage of iron could be released from the proteins under the effect of the intestinal proteinases. The iron recoveries for this experiment were 88%, 76% and 91% for ICC (2.8%), ICC (2.9%) and FeSO_4_, respectively.

### 3.2. Digestibility and Solubility of the ICC under Simulated Gastric Digestion

**Figure 2:**
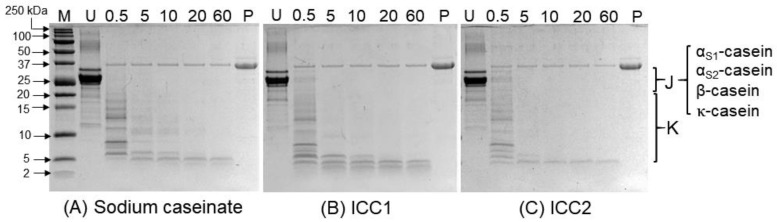
Digestibilities of (**A**) sodium caseinate and (**B**,**C**) iron‒casein complexes 1 and 2 (i.e., ICC1 and ICC2, respectively) under simulated in vitro gastric digestion over time (i.e., 0.5, 5, 10, 20 and 60 min). Samples were reconstituted in water (1%, *w/v*) and subjected to in vitro static gastric digestion at pH 2 and 37 °C using pepsin (2000 U/mL). M, molecular weight marker; U, undigested sample; P, pepsin only.

The digestibilities of the ICCs are presented in Figure 2. The undigested sodium caseinate [lane U, Figure 2A] and the undigested ICC samples [lane U, Figure 2B,C] showed all major fractions of the caseins (23.6‒19 kDa; see region J of Figure 2), indicating that their protein compositions were similar. The gels also showed some polypeptide bands eluted at molecular weights lower than that of κ-casein (i.e., 19 kDa; see region K of Figure 2), which may be attributed to the residual whey proteins in the caseinate sample.

Incubation of the samples with pepsin under simulated gastric conditions resulted in rapid hydrolysis of the proteins. All casein fractions in the sodium caseinate sample [region J, Figure 2A] showed significant hydrolysis within the first few minutes of incubation, resulting in several peptides that had molecular weights of <10 kDa [lanes 3–7 in region K, Figure 2A]. No unhydrolyzed casein fractions could be detected after 5 min of digestion [lanes 3–7 in region J, Figure 2A]. A similar pattern was observed for the ICCs over time. A comparison of the hydrolysis profiles of sodium caseinate and different ICC batches by time point is shown in Figure S1 (Supplementary Materials).

The solubilities of the ICCs and FeSO_4_ were also measured at 10, 30 and 60 min under simulated gastric digestion. The results showed that 96.5 ± 0.1, 88 ± 2.1 and 98.4 ± 0.9% of the iron from ICC (2.8), ICC (2.9%) and FeSO_4_, respectively, were soluble at 10 min. Similar solubilities were measured at 30 and 60 min.

### 3.3. In Vitro Iron Bioavailability with and without AA

**Figure 3:**
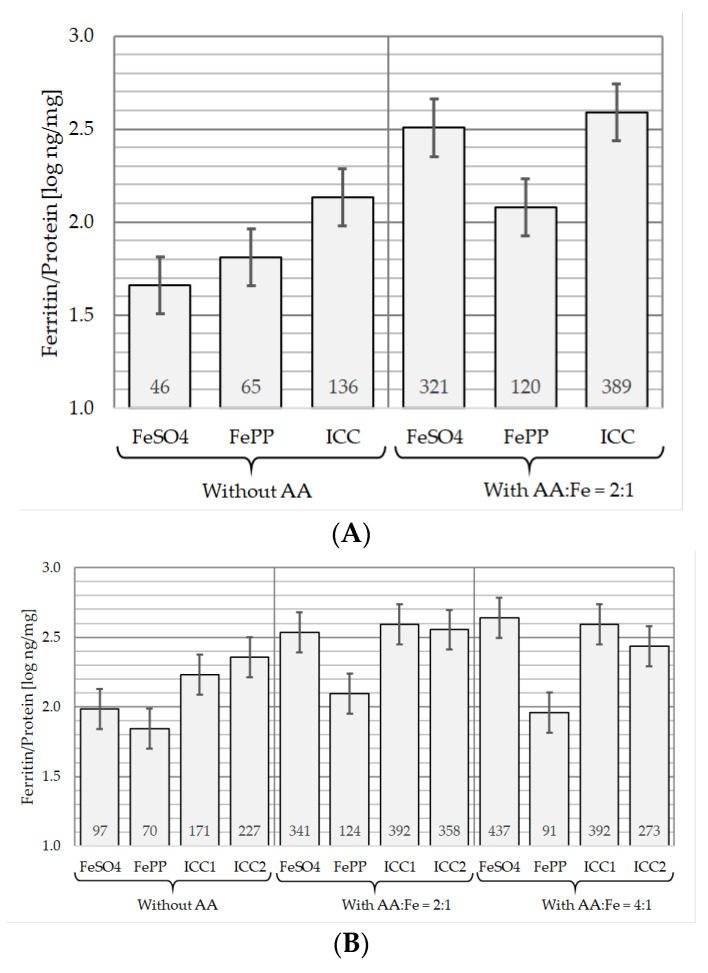
Iron uptake from ferrous sulfate (FeSO_4_), micronized ferric pyrophosphate (FePP), and iron‒casein complexes (ICCs) by Caco-2 cells after simulated in vitro digestion in water (**A**) and milk (**B**) with and without ascorbic acid (AA) (*n* = 3). In water, only ICC1 (2.8%) was tested. The two batches of ICC (i.e., 2.8% and 2.9%) were evaluated in milk. The iron was added to both drinks at a level of 3.3 mg Fe/250 mL. Molar ratios of AA to iron (Fe) are provided on the figure. The data presented are measurements of ng ferritin/mg protein after log transformation (de-log means are given on the bars). One-way analysis of variance and pairwise comparisons were performed using the Tukey‒Kramer method. Means ± 0.5 × HSD5% are presented, so that two conditions are significantly different if their error bars do not overlap.

The in vitro iron bioavailability from the ICCs was evaluated in the in vitro-simulated digestion coupled with the Caco-2 cell model with and without AA in water [Figure 3A] and in milk [Figure 3B], in comparison with FeSO_4_ and a micronized FePP.

#### 3.3.1. In the Absence of AA

As depicted in Figure 3A, in water without AA, there was a higher iron uptake by the Caco-2 cells from the ICCs than from FeSO_4_ (*p* = 0.011) and FePP (*p* = 0.037). The iron absorption from FeSO_4_ was found to be lower but not statistically different than from FePP (*p* = 0.228).

Similar results were observed from the reconstituted milk in the absence of AA for the two ICCs and FePP when considering the quantity of ferritin/mg protein produced by the cells [Figure 3B]. However, a higher iron uptake from FeSO_4_ was observed in milk, suggesting a protective effect of milk towards the rapid precipitation of FeSO_4_ observed in water without AA. Nevertheless, this was not sufficient to translate into a significantly higher iron bioavailability than from FePP in the model (*p* = 0.227).

#### 3.3.2. In the Presence of AA

As illustrated in Figure 3A, in water with the addition of AA at a molar ratio of AA to iron of 2:1, iron uptake from the three compounds by the cells was increased. The impact of AA was found to be greater for FeSO_4_ than for FePP and the ICC (i.e., ≈ 6.9-, 1.8- and 2.9-fold increase, respectively). Nevertheless, the iron uptakes by the cells from the ICC and FeSO_4_ were not statistically different (*p* = 0.473), and the in vitro iron bioavailability of both compounds was significantly higher than that of FePP (*p* < 0.01). This translates into in vitro relative bioavailability (iRBV) to FeSO_4_ of 37 and 121% for FePP and ICC (2.8%), respectively.

As shown in Figure 3B, the addition of AA to milk, at a molar ratio of AA to iron of 2:1, increased the iron bioavailability of the four compounds by ≈3.2-, 1.8-, 2.3- and 1.6-fold from FeSO_4_, FePP, ICC (2.8%) and ICC (2.9%), respectively. When compared with the data generated without AA, this enhancement was significantly different for FeSO_4_ (*p* = 0.006) and ICC (2.8%) (*p* = 0.023), but not for FePP (*p* = 0.068) and ICC (2.9%) (*p* = 0.122). Nevertheless, as in water, the addition of AA to milk increased the iron uptakes from FeSO_4_ and the two ICCs by the cells to similar levels with no statistical difference between the three compounds (*p* = 0.582; *p* = 0.852). The iron bioavailabilities from FeSO_4_ and the ICCs were significantly higher than that from FePP, translating into in vitro bioavailabilities relative to that of FeSO_4_ of 36, 114 and 104% for FePP, ICC (2.8%) and ICC (2.9%), respectively.

When AA was added to milk at a molar ratio of AA to iron of 4:1, only iron uptake from FeSO_4_ was further slightly increased by approximately an additional 1.28-fold. This did not translate into a significant difference in the model when compared with that in the presence of a molar ratio of AA to iron of 2:1 (*p* = 0.348). Under this experimental condition, the iron absorption by the cells from the two ICCs remained not statistically different from that from FeSO_4_ (*p* = 0.666, *p* = 0.113), and was found to be significantly higher than that from FePP (*p* < 0.009).

## 4. Discussion

The present publication reports on the solubility and the dissociation of iron from the ICC and on the impact of AA on the in vitro iron absorption from the complex in comparison with FeSO_4_ and FePP.

As the solubility in water or dilute acid of iron compounds used for fortification is considered to be a prerequisite for its absorption, iron salts have been classified into three categories—water soluble (e.g., FeSO_4_), poorly water soluble but soluble in dilute acid, and water insoluble and poorly soluble in dilute acid (e.g., FePP) [3,45]. In general, it is considered that water-soluble iron compounds have a high bioavailability; however, because of the inherent reactive nature of iron when fully solubilized, they can create unacceptable organoleptic changes when added to food. Conversely, the poorly soluble salts have a lower bioavailability but create fewer undesirable sensory problems in food products. Thus, whereas FeSO_4_ is the reference compound for iron bioavailability, the last category, especially FePP, is often used for the food fortification of difficult or sensitive matrices such as dairy products to avoid sensory changes [3,46,47]. Our results show that, under acidic gastric conditions, the ICC was as soluble as FeSO_4_, and the solubilities of both compounds were 2.6-fold higher than that of the tested micronized FePP. The two ICCs were also found to be digestible under simulated gastric digestion, with iron from the complexes remaining as soluble as FeSO_4_ after pepsin hydrolysis. These solubility results agree with published data on the three compounds [10,47]. A good agreement between this measurement and the relative iron bioavailability in humans has also been observed several times [10,46,47,48], especially for iron salts with good solubility in dilute acid. Therefore, although similar iron absorptions from the ICC and FeSO_4_ have already been demonstrated in humans [10], the greater solubility of the ICC when compared with FePP may be a first indication of its better in vivo bioavailability.

Whereas the solubility of inorganic salts provides information on their dissociation from their counterion, for the ICC this measurement alone is not sufficient to understand whether the ferric iron remains linked to the protein under gastric pH. The results of our in vitro experiment using size-exclusion chromatography show that most of the iron in the ICC did not dissociate from the caseins under acidic conditions (i.e., 55 to 78%). This result agrees with previous work performed with similar iron-bound casein and iron-bound caseinophosphopeptide compounds over a range of pH of 2 to 7 [25,49]. This characteristic is specific to iron chelates, such as iron bisglycinate and sodium iron EDTA (EthyleneDiaminetetraAcetic Acid). In contrast to FeSO_4_, iron chelates may be less impacted by inhibitors (i.e., phytic acid and polyphenols), especially in the absence of AA, and their relative iron bioavailability (RBV) to FeSO_4_ can be >100% in humans [3]. Therefore, the ICC may offer similar advantages, but such an effect would be observed only if the binding affinity of the caseins for iron is higher than that of the inhibitors. Another consequence of the non-dissociation of iron from casein at acidic pH is that part of the iron from the ICC may not be absorbed via the same transporter as for the inorganic salt (i.e., DMT1), as suggested for iron bisglycinate [50]. DMT1 can only mediate the active transport of the ferrous ion from the gut lumen into the enterocytes [45]. Previous work in rats, investigating the iron absorption from an iron-bound β-caseinophophopeptide, suggested that the iron absorption from this type of complex occurs via a similar pathway to that for iron gluconate, i.e., mainly energy-dependent transport that would include the transport via the DMT1. In addition, 20% of this iron-bound peptide was reported to be taken up by endocytosis [31,32]. Our experiments were not designed to investigate the mechanism of iron absorption from the ICC. However, irrespective of the means of cell entrance in the enterocytes, iron will form ferritin and a small proportion will distribute in the free iron pool [45,51], as also suggested by our Caco-2 cell experiment showing the production of similar quantities of ferritin by the cells when exposed to ICC or FeSO_4_ Finally, the limited dissociation of iron from ICC under simulated gastric conditions may also limit the impact of AA on its iron absorption. The latter has been poorly investigated on iron chelates. Only one clinical trial, aiming to evaluate the impact of AA on iron bisglycinate, was found in the literature. It showed that, in non-iron-deficient subjects (*n* = 14, mean serum ferritin 33 μg/L, range 15–74 μg/L), when added at a molar ratio of 2:1, AA increased the iron absorption from the iron bisglycinate chelate by 1.4-fold from whole cow’s milk [52]. This result suggests that part of the iron from iron bisglycinate is exchangeable in the gut lumen.

To our knowledge, the impact of AA on iron compounds such as the ICC has never been investigated. In our study, we used in vitro-simulated digestion coupled with the Caco-2 cell culture model [39,40], with FeSO_4_ and a micronized FePP as references.

In the absence of AA, iron uptake from FeSO_4_ was lower than expected and translated into a non-significant difference between the iron absorptions from FeSO_4_ and FePP. Similar results have already been reported for the Caco-2 cell model [53,54,55]. This can be explained by a rapid conversion of FeSO_4_ into the insoluble and thus non-absorbable iron hydroxide, when the pH increases to pH 7 during the in vitro digestion [35,55]. Even though comparison of the iron uptakes from FeSO_4_ in water and in milk suggested that the milk matrix may help to attenuate this effect, it was not sufficient to observe a significantly higher iron bioavailability from FeSO_4_ than from FePP with the milk condition in the model. Nevertheless, it should be noted that iron absorption from FePP in comparison with FeSO_4_ in milk without AA appears not to have been evaluated in humans. Such a comparison in a dairy matrix seems to have been made only in an iron-fortified drinkable yogurt. In this study, no significant difference in the fractional iron absorption between a micronized dispersible FePP (MDFP) and FeSO_4_ (*n* = 10, serum ferritin: 26.4 ± 21.4 μg/L; mean ± SD, range 7.9–79.3 μg/L) was observed. This result was attributed to the low particle size of MDFP and thus to its potential increased solubility (solubility data were not provided) [38]. The significantly higher iron uptake from the ICC in water suggests that the products of the enzymatic degradation of the caseins keep the undissociated iron soluble, protecting it from precipitation and thus favoring its absorption by the cells. In milk, the in vitro iron bioavailabilities from the two ICCs were found to be similar or higher compared with that from FeSO_4_, and significantly higher than that from FePP. The higher iron uptake from ICC (2.9%) than from FeSO_4_ would be due to the lower iron uptake from FeSO_4_ than expected for the reasons provided above. The results for ICC (2.8%) in comparison with FeSO_4_ agree with human data, which show no difference in iron bioavailability from the ICC and FeSO_4_ [10], and a higher bioavailability from the ICC than from FeSO_4_ in milk is not expected in humans in the absence of AA.

When AA was added at a molar ratio of 2:1, the iron uptakes from FeSO_4_ and FePP in milk by the cells were increased by 3.2- and 1.8-fold, respectively. This agrees with the retrieved human data for the same food matrix. An increased iron absorption by a factor of from 2 to 4 from FeSO_4_ in milk was reported with the addition of AA (molar ratio 2:1 or 4:1) by Walczyk et al. [16]. Pauline et al. [14] showed that fortified milk with an equimolar amount of AA increased the fractional iron absorption from FePP by almost 2-fold. Therefore, the 1.8-fold increase in iron uptake from FePP in the model would reach a significant difference in humans, but the absorption may remain low when compared with other iron compounds, especially in iron-deficient subjects [56]. Similar ranges of enhancement for FeSO_4_ have also been reported from other food matrices [37]. AA increased the in vitro iron bioavailabilities from the ICCs in milk by 1.6- to 2.3-fold. This matches with the 1.4-fold increase reported for the iron bisglycinate chelate in milk [52]. Although the low impact of AA on the in vitro iron bioavailability from FePP is probably due to its low solubility under acidic or gastric conditions (i.e., ≈37% after 90 min versus >85% for the ICCs), the lower impact of AA on the ICC chelates than on FeSO_4_ can be explained by the extent of dissociation of iron from the complex, limiting its exchange with AA. However, it is important to emphasize that iron uptake from the ICCs by the cells was not significantly different from that from FeSO_4_, indicating that the iron that did not dissociate under acidic pH and thus that did not interact with AA during the simulated intestinal digestion was bioavailable. The calculated relative in vitro bioavailabilities (*i*RBV) of FePP, ICC (2.8%) and ICC (2.9%) to that of FeSO_4_ were 36%, 114% and 104%, respectively, in milk with added AA. In humans, the RBV of FePP to FeSO_4_ in milk with the same molar ratio of AA to iron is reported to be 32% [*n* = 20, serum ferritin 17.8 (5.8–67.2) μg/L [geometric mean (range)]] [47]. The observed correlation between the *i*RBV of FePP calculated from the model and the reported RBV as determined in humans with a rather low iron status suggests that the model can be used to estimate the RBV in human settings with high requirements for iron. It is recognized that the lower the iron status of an individual (as defined by serum ferritin), the higher the iron absorption will be [11]. However, the upregulation of iron absorption of some iron salts, such as FePP, has been reported to be limited in subjects with low iron status when compared to FeSO_4_, impacting its RBV to FeSO_4_ [36,56]. This may be due to its poor solubility under acidic gastric conditions. In a previous human study, Henare et al. [10] showed no significant difference between the slope of linear regression of fractional iron absorption and serum ferritin concentration of ICC and FeSO_4_ and concluded that it would be expected that the RBV of the ICC to FeSO_4_ would be consistent across individuals of different iron status. Therefore, considering its solubility and the present Caco-2 cell results, the ICC would be expected to exhibit a similar RBV in humans as measured in single-meal studies than that measured in the cell model (i.e., with a molar ratio of 2:1 in milk), since in addition the model has been shown to accurately predict the human response to the interaction of AA with iron absorption [40].

When AA was added to milk at a molar ratio of AA to iron of 4:1, only the iron absorption of FeSO_4_ was further slightly increased by an additional 1.28-fold in the model. Interestingly, a similar enhancement (i.e., by 1.18-fold *p* < 0.05) was reported by Walczyk et al. [16] in a study evaluating the iron absorption from milk containing various levels of calcium and AA (3 mg of iron per serving) in children (6–11 years) (*n* = 32 per group, 50% iron repleted‒50% with iron deficiency anemia), showing again an agreement between the results from the Caco-2 cell model and the human data. The iron absorptions were 32.3% (18.4; 56.5) and 38.1% (22.1; 65.5) (geometric mean −SD; +SD) from milk, with the addition of AA at molar ratios of 2:1 and 4:1, respectively [16]. The lack of an additional effect from the increased amount of AA on the ICC and FePP in the model indicates that there was no remaining iron in the digesta that could be exchanged with AA. Under this experimental condition, iron uptake from the two ICCs remained not statistically different from that from FeSO_4_, and the iron absorptions from these three compounds by the cells were found to be significantly higher than that from FePP. Thus, even though the higher molar ratio of AA to iron was not found to have an additional effect on the ICC in the model, in humans, it would still be expected to have a higher absorption than FePP under similar conditions. However, a higher absorption from FeSO_4_ with this increased level of ascorbic acid (i.e., to 4:1 molar ratio) cannot be excluded. It should be noted that the addition of AA at a molar ratio of 4:1 is recommended only for food matrices that contain high levels of phytic acid, such as cereals [3].

## 5. Conclusions

The ICC was found to be as soluble as FeSO_4_ under simulated gastric digestion. As part of its iron did not dissociate from the casein under acidic conditions, the ICC can be classified as an iron chelate. The demonstrated impact of AA on iron uptake from the ICC showed that part of the iron that dissociates under simulated digestion is exchangeable in the gut lumen; also, the similar quantity of ferritin generated by the cells when exposed to ICC and FeSO_4_ would suggest that the part of iron from ICC that is not dissociated in lumen is further processed in the cell to allow the formation of ferritin. Finally, the ICCs were absorbed in a similar amount to FeSO_4_ in the presence of AA, and in a significantly higher amount than FePP with and without AA at the molar ratio of AA to iron recommended by the World Health Organization for an optimal iron absorption from iron-fortified milk. Therefore, with its improved organoleptic properties when compared with highly soluble inorganic iron salts, the ICC may become a compound of choice for the iron fortification of dairy products.

## Supplementary Materials

The following are available online at https://www.mdpi.com/2072-6643/12/9/2776/s1, Figure S1: Digestibility of sodium caseinate and iron‒casein complexes under simulated in vitro gastric digestion at 5, 10, and 60 min.
