# Supplementary material for: Impact of Ascorbic Acid on the In Vitro Iron Bioavailability of a Casein-Based Iron Fortificant
Source: Nutrients. 2020 Sep 11;12(9):2776. doi: 10.3390/nu12092776 (PMC7551990; doi:10.3390/nu12092776)
Supplement: Supplementary file 1 [file nutrients-12-02776-s001.pdf]

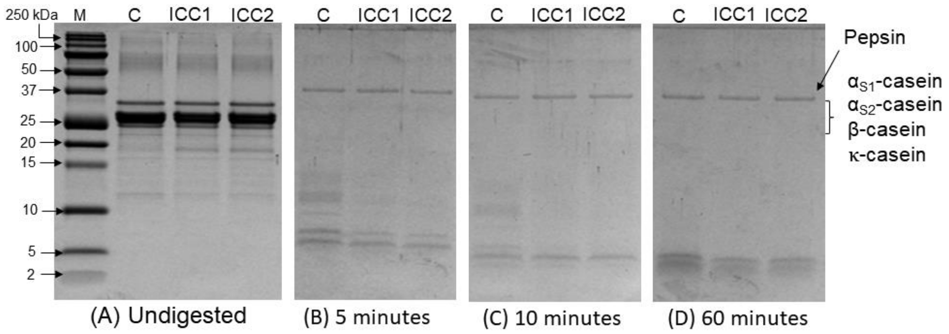

Figure S1. Digestibility of sodium caseinate and iron–casein complexes under simulated in vitro gastric digestion at 5, 10, and 60 min. A represents undigested samples while B-D represents hydrolysis profiles after 5, 10 and 60 minutes of digestion. Samples were reconstituted in water (1% w/v) subjected to static digestion at pH 2 and 37°C using pepsin (2000 U/mL). M, molecular weight marker; C, sodium caseinate; ICC1 and ICC2 indicate different batches of iron-casein complexes.
